# Evaluation of the factors affecting the prognosis of patients with solid tumors and hematologic malignancies in the intensive care unit

**DOI:** 10.1186/2197-425X-3-S1-A245

**Published:** 2015-10-01

**Authors:** Y Savran, R Arikan, B Comert

**Affiliations:** Internal Medicine Intensive Care Unit, Dokuz Eylul University Faculty of Medicine, Izmir, Turkey; Internal Medicine, Dokuz Eylul University Faculty of Medicine, Izmir, Turkey

## Intr

Cancer has an increasing incidence worldwide and has a high mortality rate. Patients diagnosed with cancer need to be admitted to intensive care units for various reasons. As a result of the shortage of beds in intensive care units, physicians feel reluctant about the admission of these patients to intensive care units.

## Objectives

This study aimed to show the prognostic factors of the patients with solid tumors and hematologic malignancies in intensive care units, and hence guide physicians to manage patients with cancer and their intensive care units.

## Methods

This study included patients who were hospitalized in Dokuz Eylul University Hospital Internal Medicine Intensive Care Unit for more than 24 hours during tJanuary 2012-December 2013 interval. The patients were divided into three groups; patients with solid tumors, patients with hematologic malignancies, and patiens without cancer. Their data was analyzed retrospectively. All patients´ data (demographic data, organ failure, comorbid diseases, critical diseases, presence of infection, immunsupressive treatments within the last month) were recorded at the time of admittance.Additionally presence of remission, history of bone marrow transplantation, previous treatments, presence of neutropenia of patients with solid tumors and hematologic malignancies were also recorded.

## Results

Mortality rate in intensive care unit was found to be %46.0 in general, %68.6 in patients with solid tumor, %53.0 in patients with hematologic malignancy, and %39.8 in the control group (n = 512; solid tumor = 89, hematologic malignancy = 49, control group = 374). The mortality rate was especially high among patients with solid tumors. In the logistic regression model, APACHE II score and the need of invasive mechanical ventilation at the time of admission to intensive care unit were found as independent risk factors for increased mortality. In addition, the need of renal replacement therapy at the time of admission to intensive care unit was found as an independent risk factor for increased mortality among the control group.

## Conclusions

The mortality rate of patients in intensive care units is high, especially those with solid tumors. At the time of admission to the intensive care unit, for cancer patients with critical illness, the APACHE II score and the need of invasive mechanical ventilation should be evaluated, for these are strong predictors of increased mortality.
